# External validation of the colorectal cancer risk score LiFeCRC using food frequency questions in the HUNT study

**DOI:** 10.1007/s00384-024-04629-4

**Published:** 2024-04-25

**Authors:** Siv S. Brenne, Eivind Ness-Jensen, Eivor A. Laugsand

**Affiliations:** 1 Department of Surgery, Levanger Hospital, Nord-Trøndelag Hospital Trust, Levanger, Norway; 2 HUNT Research Centre, Department of Public Health and Nursing, NTNU, Norwegian University of Science and Technology, Forskningsveien 2, N-7600 Levanger, Norway; 3 Department of Medicine, Levanger Hospital, Nord-Trøndelag Hospital Trust, Levanger, Norway; 4 Upper Gastrointestinal Surgery, Department of Molecular Medicine and Surgery, Karolinska Institutet, Karolinska University Hospital, Stockholm, Sweden

**Keywords:** Colorectal cancer, Risk, Cancer screening, Colon, Rectal

## Abstract

**Purpose:**

To mitigate the increasing colorectal cancer (CRC) incidence globally and prevent CRC at the individual level, individual lifestyle information needs to be easily translated into CRC risk assessment. Several CRC risk prediction models exist and their clinical usefulness depends on their ease of use. Our objectives were to assess and externally validate the LiFeCRC score in our independent, unselected population and to investigate the use of simpler food frequency measurements in the score.

**Methods:**

Incidental colon and rectal cancer cases were compared to the general population among 78,580 individuals participating in a longitudinal health study in Norway (HUNT). Vegetable, dairy product, processed meat and sugar/confectionary consumption was scored based on food frequency. The LiFeCRC risk score was calculated for each individual.

**Results:**

Over a median of 10 years following participation in HUNT, colon cancer was diagnosed in 1355 patients and rectal cancer was diagnosed in 473 patients. The LiFeCRC score using food frequencies demonstrated good discrimination in CRC overall (AUC 0.77) and in sex-specific models (AUC men 0.76 and women 0.77) in this population also including individuals ≥ 70 years and patients with diabetes. It performed somewhat better in colon (AUC 0.80) than in rectal cancer (AUC 0.72) and worked best for female colon cancer (AUC 0.81).

**Conclusion:**

Readily available clinical variables and food frequency questions in a modified LiFeCRC score can identify patients at risk of CRC and may improve primary prevention by motivating to lifestyle change or participation in the CRC screening programme.

**Supplementary Information:**

The online version contains supplementary material available at 10.1007/s00384-024-04629-4.

## Introduction

Nearly half of all cancer cases globally are attributable to modifiable risk factors [1]. Colorectal cancer (CRC) has been identified as being among the most preventable cancers, by being attributable to certain behavioural, occupational, metabolic and life-style related risk factors [2, 3]. CRC has an expected rise in global burden of disease of 60%, reaching 2.2 million new cases and 1.1 million deaths in 2030 [4]. This increase necessitates improved strategies for primary prevention. Although CRC screening is implemented in most of the countries where CRC is highly prevalent, the participation rates in screening programmes remain variable (faecal occult blood tests 7–90% and sigmoidoscopy 7–55%), and mostly far from the desired rates [5]. It has been suggested that individualised CRC risk assessment methods, with risk stratification to identify high-risk individuals, may aid more targeted prevention in the asymptomatic population, increase the participation in screening and motivate for lifestyle modification at an individual level, as well as be a cost-effective approach to reduce the burden of CRC [6].

Several risk prediction models for CRC have been developed [6–10]. Many of these models were created using data from case–control studies prone to recall bias [11]. The LiFeCRC score is to our knowledge the most recent CRC risk prediction model for Europe [12]. It was based on data from 255,482 participants in the European Prospective Investigation into Cancer and Nutrition (EPIC) and demonstrated good discriminative ability with Harrell’s C-index 0.710 in the derivation cohort and 0.714 in the validation cohort [12]. It has been stated that further scrutiny of prediction models is warranted, as there is variability in the eligibility criteria used and external validation is lacking [6]. For instance, the abovementioned model excluded individuals > 70 years old and patients with diabetes [12]. Furthermore, growing evidence supports that the risk profile for cancer in the colon differs from that in the rectum and may also differ for men and women [13, 14]. Hence, risk prediction models developed for CRC need to be externally validated for these subgroups.

The clinical usefulness of CRC risk prediction models depends on their ease of use. While sex, age, weight, height and smoking history may be easily obtained, other lifestyle and dietary-related factors such as consumption of different kinds of food and alcohol may be more challenging. As an alternative to measurements of food intake in grammes/day, food frequency questionnaires (FFQs) are an established assessment method often used when investigating the effects of diet on disease outcomes [15, 16]. Recently, time-efficient FFQs have demonstrated a good ability to estimate intakes of different kinds of foods in colorectal cancer patients [17, 18].

Our objective is to assess and externally validate the LiFeCRC score in an unselected population and to investigate the use of simpler food frequency measurements in the score.

## Materials and methods

### Study population

This is a prospective cohort study, based on the Trøndelag health study (HUNT) conducted in Nord-Trøndelag County (Norway) in four surveys (HUNT1 (1984–1986), HUNT2 (1995–1997), HUNT3 (2006–2008) and HUNT4 (2018–2019). All residents aged > 20 years were invited. At local assessment centres, participants completed extensive questionnaires on sociodemographic characteristics, medical, dietary and lifestyle factors (including, but not limited to smoking, alcohol consumption, physical activity) at baseline. Completed questionnaires, standardised clinical measurements done by trained personnel (such as blood pressure, weight, height, hip and waist circumference) and blood samples from all participants were collected and stored in the HUNT biobank [19]. Participants in HUNT2 (*n* = 65,237, 69% of the entire population) and/or HUNT3 (*n* = 50,807, 54% of the entire population) were eligible for the present study.

### Definition of outcome

**Fig. 1:**
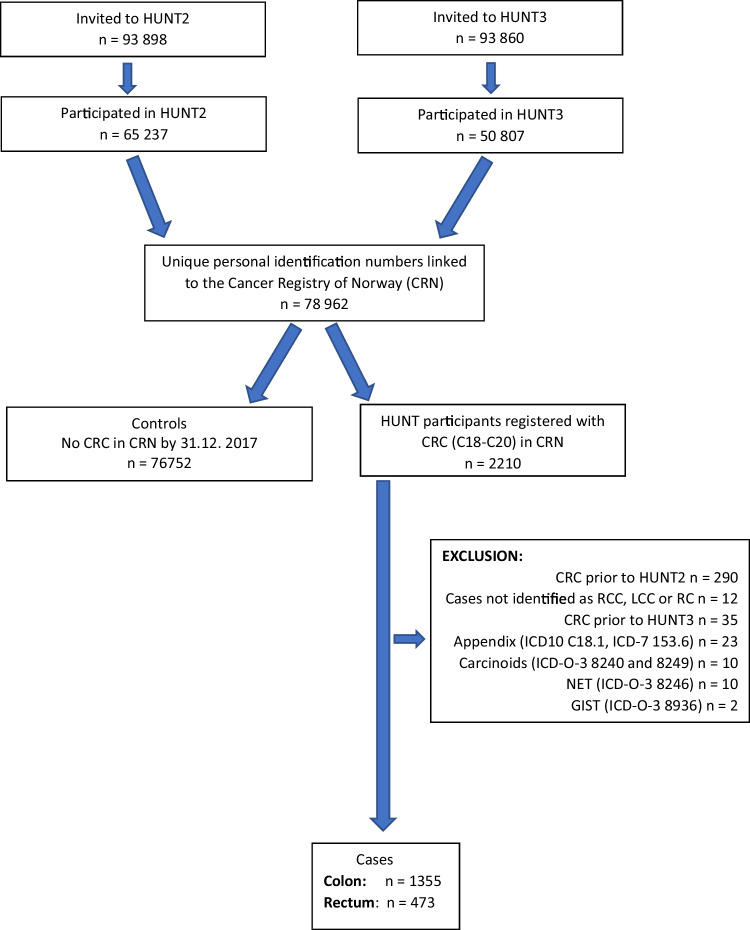
Flow-chart of cases and controls

The primary outcome was the first diagnosis of colon cancer registered in the Cancer Registry of Norway (CRN), after participation in HUNT2 and/or 3 until the end of follow-up 31st of December 2017. Linkage between the CRN and HUNT was possible due to the unique national personal identification number assigned to each Norwegian inhabitant. Reporting of data to CRN is mandatory by law for all institutions diagnosing and treating cancer patients in Norway. Colon cancer cases were identified according to the International Classification of Diseases, 7th edition (ICD-7) codes 153.0 and 10th edition (ICD-10) codes C18-19 (excluding C18.1 Appendix), and the morphological codes according to the International Classification of Diseases for Oncology, 3rd edition (ICD-O-3) codes 8041, 8144, 8210, 8211, 8255–8263, 8480–8481, 8490, 8510, 8570–8574, 6900, 6999, 8000–8020 (excluding carcinoids 8240 and 8249, neuroendocrine tumours 8246 and gastrointestinal stromal tumours 8936). Right colon cancer (RCC) was defined as ICD7 153.0–153.1, left colon cancer (LCC) was defined as ICD7 153.2–153.4. For synchronous cancers not located in the same subsegment (*n* = 7), the most distal cancer localisation was used. The controls were defined as those participating in HUNT2 and/or HUNT3 who were not diagnosed with colorectal cancer in the Cancer Registry of Norway (1956 through 2017). The cancer stage was recorded based on data from hospital journals according to the tumour, node, metastasis (TNM) classification [20]. A flowchart of cases and controls is shown in Fig. 1.

### Definition of predictors

All predictors included in the established LiFeCRC risk prediction models were considered potential predictors in the present analysis and preferentially handled as in the original LiFeCRC score publication with regard to being continuous or categorical [12]. Alcohol intake was measured as daily intake (yes or no) in the original LiFeCRC publication [12], whereas it was measured as “ ≥ once/week” or “less” in HUNT. Smoking was categorised as yes if current or former smoker and as no if never smoker. Based on the questions about various types of physical activity, the metabolic equivalents (METs) and the MET hours/week were calculated as in previous HUNT publications [21]. The individuals were categorised as physically active or not according to the calculated MET hours/week (physically active “yes” > 8.3 MET-h/week or “no” ≤ 8.3 MET-h/week) (Supplementary Table 1). In HUNT, dietary information was collected in semi-quantitative food frequency questionnaires (FFQs). The validity and reproducibility of dietary patterns and food consumption assessed in FFQs have been documented previously [15, 16, 22]. The food frequency questionnaire variables in HUNT were translated into g/day as required by Aleksandrova et al., based on the Norwegian food-based dietary guidelines (FBDG) [23, 24]. The risk factors and their definitions are presented in Supplementary Table 1.

### Statistical analyses

Characteristics of the study population are presented as proportions for categorical variables and as means with standard deviations (SD) for continuous variables. Comparison between subgroups was made by using the independent samples *t*-test for continuous variables and the chi-square test for categorical variables. We used available case analysis, and no imputations were made. That is, we included all subjects with data on the relevant variables, and the percentages refer to the share of available answers. The risk of colorectal, colon and rectal cancer was assessed using multivariable Cox regression analysis with observational time defined from participations in HUNT2 or HUNT3 until cancer diagnosis or end of follow-up 31st December 2017. Hazard ratios (HRs) with 95% confidence intervals (CIs) were reported separately for these three subgroups, and separately for men and women. For cases participating in both HUNT2 and 3, the survey closest prior to the cancer diagnosis was chosen as a baseline. For controls participating in both HUNT2 and 3, HUNT3 was chosen as exposure time as the questionnaires in HUNT3 were more comprehensive.

The individual LiFeCRC risk score was calculated with the external weights as previously published [12]:

$$\begin{aligned}\text{Risk Score}_{\text{i}} = 0.0781 &\times \text{Age}_{i} \;(\text{years})\\&+0.0117 \times \text{Waist circumference}_{i}\; (\text{cm})\\&+0.0115 \times \text{Body height}_{\text{i}} \;(\text{cm})\\&+0.1292 \times \text{Weekly alcohol}_{\text{i}} \;(\text{yes} = 1,\, \text{no} = 0)\\&+0.2125 \times \text{Smoking}_{\text{i}}\; (\text{yes} = 1,\, \text{no} = 0)\\&-0.0964 \times \text{Physically active}_{\text{i}}\; (\text{yes} = 1,\, \text{no} = 0)\\&-0.0773 \times \text{Vegetable intake}_{\text{i}} \;(\text{food frequency})\\&-0.0166 \times \text{Dairy products intake}_{\text{i}}\; (\text{food frequency})\\&+0.0808 \times \text{Processed meat intake}_{\text{i}}\; (\text{food frequency})\\&+0.0268 \times \text{Sugar and confectionary}_{\text{i}}\; (\text{food frequency})\end{aligned}$$

The risk score models for colorectal, colon and rectal cancer, as well as separate models for men and women, were plotted as receiver operating characteristic (ROC) curves. Area under the ROC curves (AUC with 95% confidence interval) were estimated for each of the models. The model calibration was assessed graphically by plotting observed risk against the predicted risk of developing CRC over the next 10 years. The individual 10-year absolute risk for CRC was estimated by the previously published formula integrating the calculated LiFeCRC Risk Score_i_, and these values were divided into deciles with computed means and standard deviations [12]. For each decile group of predicted risk, the Kaplan-Meier survival function at 10 years was calculated with 95% confidence intervals for CRC, colon cancer, and rectal cancer, for both sexes combined as well as for men and women separately. Analyses estimating sensitivity, specificity, positive and negative predictive values for the high risk (> 90th percentile) and high or intermediate risk groups (between the 50th and the 90th percentile), as defined by the previously published cut points for 10 year- absolute risk of CRC, were calculated for all participants, both sexes collected as well as men and women separately [12]. The statistical software SPSS for Windows version 29.0 and GraphPad Prism version 10 was used for all statistical analyses.

### Ethics

All participants in HUNT gave a written informed consent, including consent for linkage to their medical records as well as other central health registers in Norway. In addition, a specific ethical approval for this study was obtained from the ethical review committee. All data was stored and handled confidentially.

## Results

### Characteristics

Over a median of 10 years following participation in HUNT, colon cancer was diagnosed in 1355 patients and rectal cancer was diagnosed in 473 patients. The characteristics of cases and controls are presented in Table 1. Among the 78,580 participants, there were 41,754 women (53%). The cases were generally older than the controls (mean age of cases 66 years and of controls 53 years), had longer waist circumference, higher BMI, smoked more, were less physically active and more had diabetes (*p* < 0.001) (Table 1).

**Table 1:** Characteristics of the study population (*N* = 78,580)

	Cases	Controls	*p*	Missing *n* (%)
Total, *n* (%)	1828 (2.3)	76,752 (97.7)		0
Female sex, *n* (%)	897 (49.1)	40,857 (53.2)		
Subgroups, *n* (%)				
Right colon cancer	763 (41.8)			0
Left colon cancer	591 (32.2)			0
Rectal cancer	473 (25.9)			0
Age at recruitment, years, mean (SD)	65.7 (11.7)	53.1 (17.8)	** < 0.001**	0
Waist circumference, cm, mean (SD)	93.5 (12.0)	91.5 (12.6)	** < 0.001**	905 (1.2)
Height, cm, mean (SD)	169.0 (9.3)	170.2 (9.5)	** < 0.001**	1036 (1.3)
BMI, kg/m^2^ mean (SD)	27.6 (4.2)	26.9 (4.4)	** < 0.001**	779 (1.0)
Smoking, packyears, mean (SD)	10.9 (14.3)	7.5 (11.6)	** < 0.001**	9996(12.7)
Physical activity, MET-h/week, mean (SD)	8.4 (7.6)	10.6 (8.9)	** < 0.001**	15,933(20.3)
Diabetes, *n* (%)	125 (6.9)	3536 (4.6)	** < 0.001**	119 (0.2)
Alcohol, ≥ once a week, *n* (%)	274 (15.0)	18,059 (23.5)	** < 0.001**	1437 (1.8)
Vegetables, < daily intake, *n* (%)	334 (45.0)	25,452 (51.3)	** < 0.001**	28,176 (35.9)
Milk, < 1 glass/day, *n* (%)	230 (17.1)	10,462 (16.5)	0.555	13,672 (17.4)
Processed meat, ≥ daily intake, *n* (%)	7 (0.8)	155 (0.3)	**0.018**	30,476 (38.8)
Chocolate, ≥ daily intake, *n* (%)	36 (4.2)	2524 (5.4)	0.134	30,648 (39.0)
Bread, dark/crisp, *n* (%)	938 (99.6)	64,489 (98.8)	**0.032**	12,378 (15.8)
Fruit/berries, < daily intake, *n* (%)	330 (44.5)	24,087 (48.5)	**0.029**	28,183 (35.9)
Fish, < daily intake, *n* (%)	900 (96.6)	47,515 (98.2)	** < 0.001**	29,256 (37.2)

Proportions of categorical variables (% based on complete cases) and means with standard variations for continuous variables. Comparisons made by chi-square tests for categorical variables and independent samples *t*-test for continuous variables. Data in bold emphasis indicate *p*<0.05

*Cm* centimetres, *SD* standard deviation, *MET-h/week* metabolic equivalent hours/week, *BMI* body mass index in kilogrammes/metre^2^

### The possible predictors of CRC overall, of colon and rectal cancer

Age is the most consistent risk factor throughout all analyses performed, increasing the risk of CRC by 8% per year for men and women (*p* < 0.001, Supplementary Table 2). Waist circumference, height, alcohol consumption and smoking were all significantly associated with the combined outcome CRC when both sexes were analysed together (*p* < 0.05, Supplementary Table 2). In the separate analyses for men and women, waist circumference was a significant predictor only in men (*p* < 0.01), whereas smoking was a significant predictor only in women (*p* < 0.05, Supplementary Table 2). In colon cancer sub-analyses, waist circumference, height and smoking were significant predictors when both sexes were analysed together (Supplementary Table 3). Only waist circumference remained significant in analyses of colon cancer in men and no other factors than age were significant predictors in women (Supplementary Table 3). In rectal cancer sub-analyses, age was the only significant predictor (*p* < 0.001, Supplementary Table 4).

### Validation of the LiFeCRC risk score

**Fig. 2:**
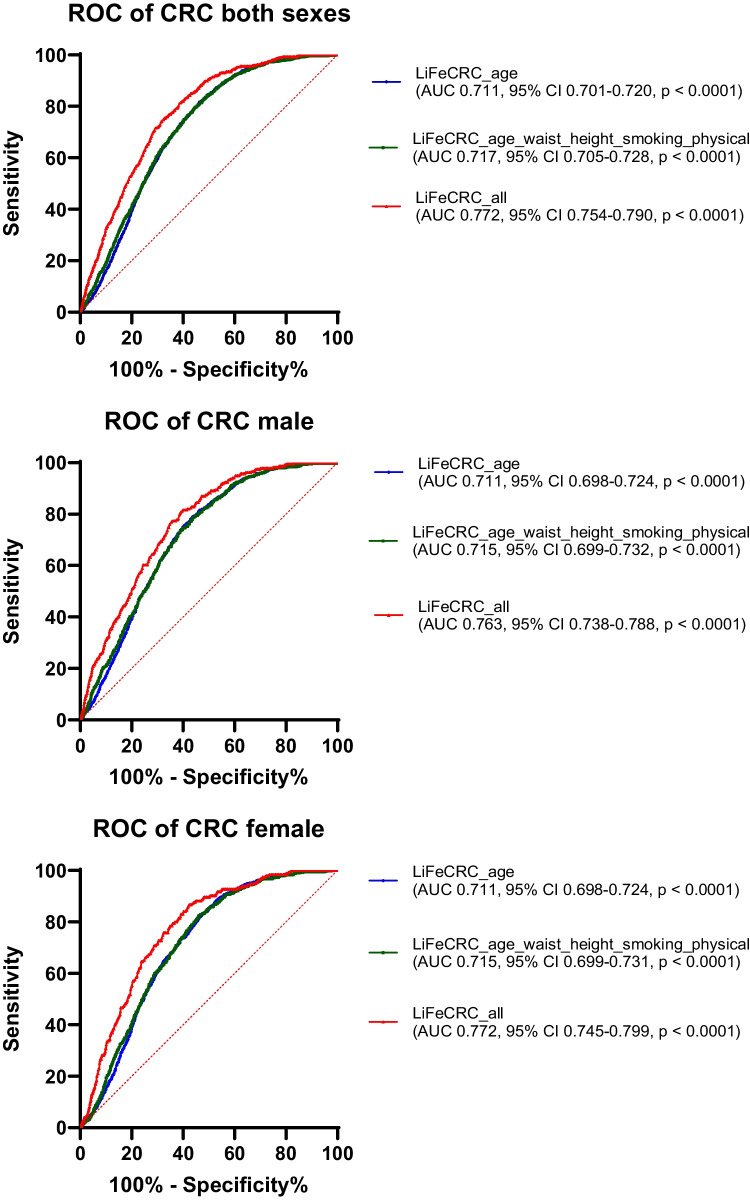

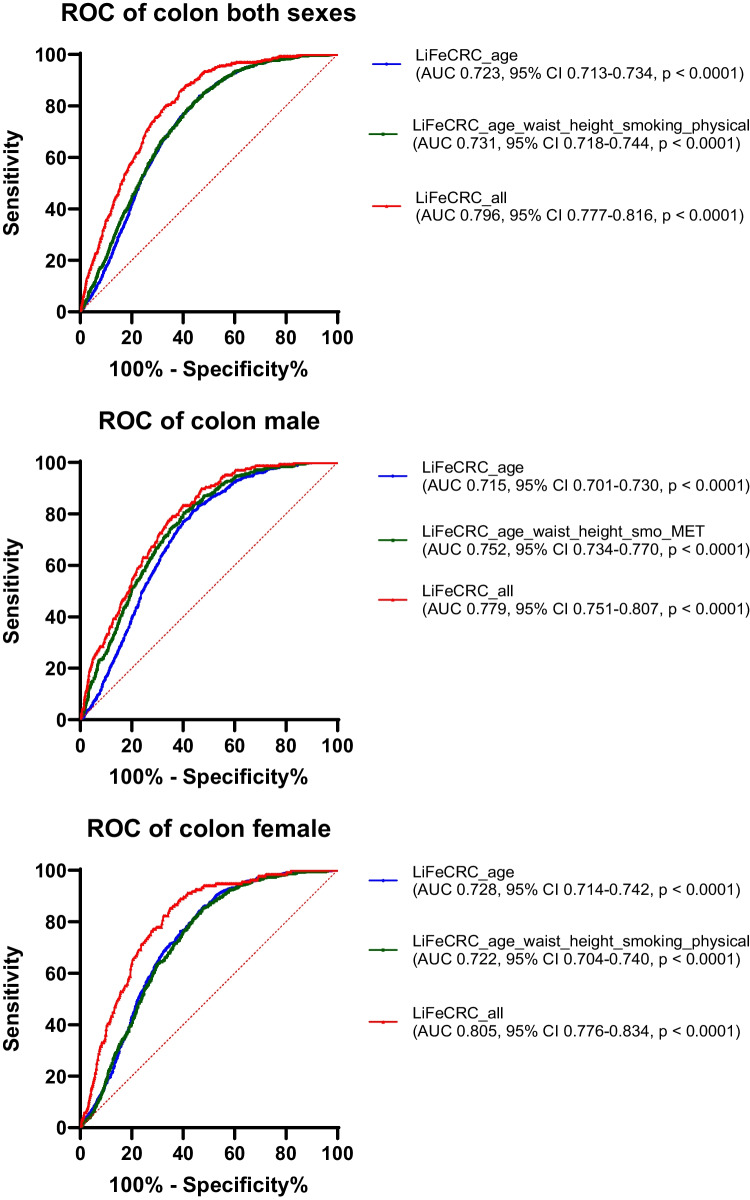

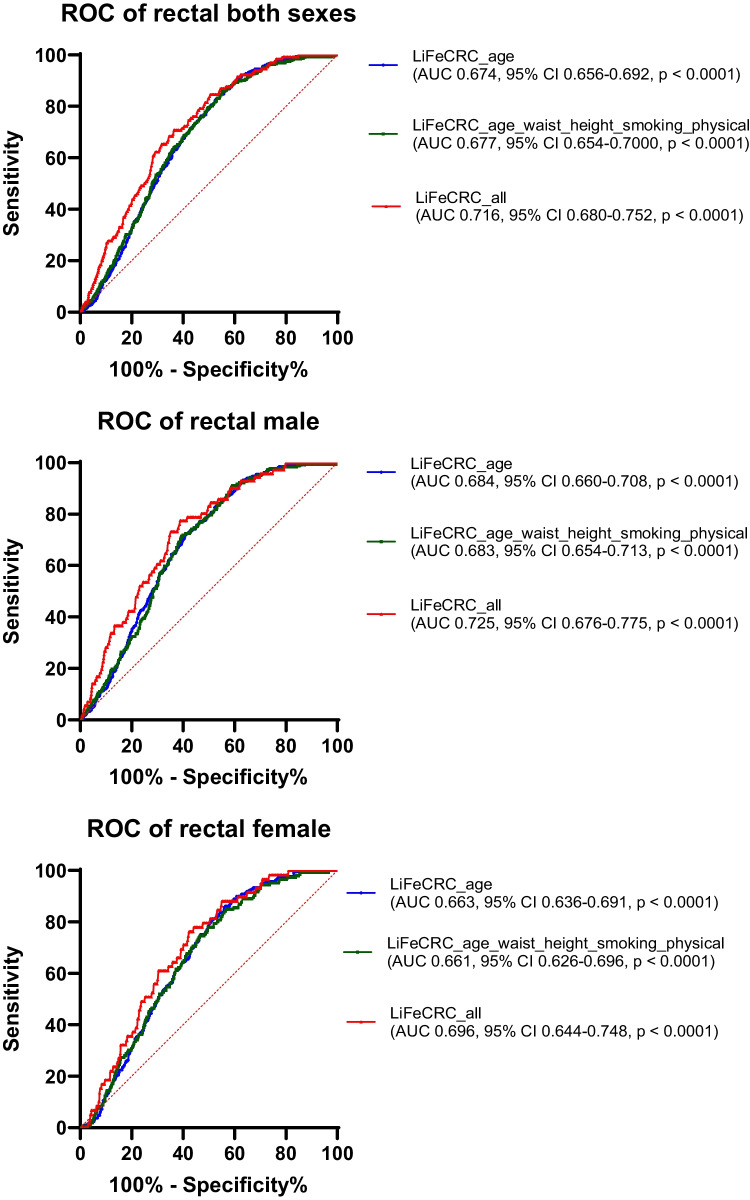
ROC curves illustrating discrimination of the risk score models for colorectal, colon and rectal cancer, as well as separate models for men and women. Area under the curves (AUC) with 95% confidence interval (CI) were estimated for each of the models

**Fig. 3:**
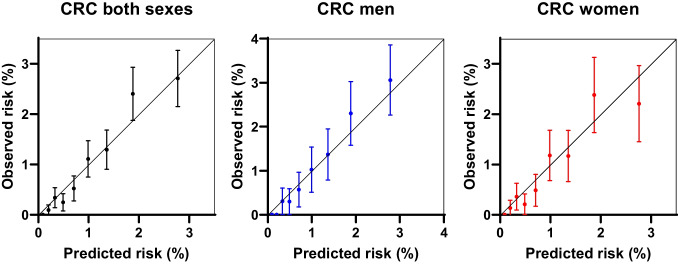
Calibration assessed graphically by plotting observed risk against predicted risk of developing CRC over the next 10 years in both sexes, men and women. Predicted risk is compared against observed risk based on the complement of the Kaplan–Meier survival curve

ROC curves of the LiFe scores including only age (blue), then age and the lifestyle-based measurements, waist circumference, height, smoking and physical activity (green) and finally also including intake of alcohol, vegetables, dairy products, processed meat and sugar/confectionary (red) were plotted to better visualise the contribution from the subsets of factors (Fig. 2). As demonstrated by all the ROC curves in Fig. 2, age is the main risk prediction factor. The calculated LiFeCRC risk score, implementing the use of food frequencies, demonstrated good discrimination in CRC overall (AUC 0.77) and in sex-specific models (AUC men 0.76 and women 0.77) in this population, also including individuals ≥ 70 years and patients with diabetes (Fig. 2). It performed somewhat better in colon (AUC 0.80) than in rectal cancer (AUC 0.72) (Fig. 2). Model calibration is illustrated in Fig. 3 and demonstrates good calibration for CRC overall and in sex-specific analyses. Table 2 shows sensitivity, specificity and positive and negative predictive values for the high-, as well as high- or intermediate-risk groups. Sensitivity was consistently higher in men than in women both for CRC overall, and for colon and rectal cancer when evaluated separately. Specificity for both colon and rectal cancer was above 87% for both men and women in the highest risk group (Table 2). The negative predictive value (NPV) was > 98% throughout all subanalyses (Table 2).

**Table 2:** Sensitivity, specificity, positive and negative predictive values

	CRC			Colon			Rectum		
Risk level*	Both sexes	Men	Women	Both sexes	Men	Women	Both sexes	Men	Women
**High**									
Sensitivity	31.3	36.4	25.1	34.2	37.5	30.1	24.6	33.8	13.6
Specificity	90.3	87.4	92.5	90.3	87.4	92.5	90.3	87.4	92.5
PPV (%)	4.2	4.7	3.5	3.2	3.4	3.0	1.0	1.3	0.6
NPV (%)	99.0	98.8	99.1	99.3	99.2	99.4	99.7	99.6	99.7
**High or IM**									
Sensitivity (%)	90.3	93.3	86.7	93.4	95.2	91.2	83.1	88.7	76.3
Specificity (%)	50.5	42.7	56.7	50.5	42.7	56.7	50.5	42.6	56.7
PPV (%)	2.4	2.7	2.1	1.8	1.9	1.6	0.7	0.8	0.6
NPV (%)	99.7	99.7	99.7	99.9	99.9	99.9	99.8	99.9	99.9

*CRC* colorectal cancer, *PPV* positive predictive value, *NPV* negative predictive value

*Risk groups are based on predicted 10-year absolute risks of colorectal cancer using the originally published LiFeCRC score, i.e. participants are categorised as *low risk* when below the 50th percentile (0.84%), *high risk* when above the 90th percentile (3.54%) and *intermediate (IM) risk* when between the 50th and 90th percentiles

## Discussion

In this study, we externally validated the LiFeCRC score. We demonstrated that the model performance is good (AUC > 0.70), also in a population including individuals ≥ 70 years and patients with diabetes. The model showed good discrimination properties for both colon cancer and rectal cancer and in sex-specific sub-analyses. The prediction model works well with responses to food frequency questions as the model discrimination was higher in the model implementing food frequency questions (AUC 0.77 for CRC in both sexes) than that of the original LiFeCRC publication (AUC 0.71 for CRC in both sexes) [12]. Throughout all subanalyses, the model including food frequency questions had better discrimination capacity than using only age as a predictor. By using the full model including the responses to food frequency questions, approximately one-third and 90% of the patients developing CRC were identified among the 10% and 50% with the highest score, respectively. Altogether, the modified LiFeCRC score may be a relevant tool in personalised CRC screening and communication of individual CRC risk and prevention.

The simple LiFeCRC risk score is to our knowledge not routinely used for risk assessment in any asymptomatic European population. Factors hampering its clinical use may be that measurements of food intake in g/day are considered difficult for patients to recall and the fact that questions about daily alcohol consumption are perceived as sensitive in many countries. In our modified LiFeCRC risk score, we demonstrated that answers to food frequency questions perform at least as good as the g/day measurements. In the original LiFeCRC risk score, alcohol intake was scored as daily (yes or no) and the hazard ratio for CRC in men was 1.18 (95% CI 1.06–1.30) [12]. In another risk score publication, alcohol intake was scored as weekly (yes or no) and the odds ratio for colorectal cancer in men was 1.36 (95% CI 1.08–1.71) [25]. In our modified LiFeCRC risk score, alcohol consumption was scored as weekly (yes or no). Possibly, the challenges with alcohol stigma may be bypassed by asking about weekly rather than daily alcohol consumption. However, further research is needed to decide upon at what level alcohol consumption is a risk factor, as previous studies have used many different cut-off values (Supplementary Table 1). By finding that the modified LiFeCRC risk score performs well in a population including individuals ≥ 70 years and patients with diabetes, we demonstrate that this risk score may be used in clinical practice for CRC risk assessment in an unselected, asymptomatic, European population.

Screening may reduce CRC incidence and mortality [26], but these effects are highly dependent on participation rates and, particularly that the individuals at high risk of CRC participate [5]. High risk of CRC has been associated with factors such as lower education levels, current smoking and lower socioeconomic status, and these individuals are typically among those not attending the CRC screening programme [5, 6, 27]. Most CRC screening programmes use a one-size-fits-all approach for inclusion, but the use of age as the only criterion to enter the programme has been criticised for not being cost-effective, as they do not reach the target population [6]. We believe that our findings, illustrating the added value of other factors than age, support the recently suggested “personalisation” strategies leading to precision CRC screening [28]. The former lack of clinically useful, validated CRC risk prediction models has been identified as one of the challenges in implementation of precision CRC screening [6].

In line with the existing literature, we found that our modified LiFeCRC risk score had equal performance for men and women [7, 12, 29, 30]. This may be an advantage if used for motivation of males to participate in screening, where males have traditionally been underrepresented [5]. Furthermore, the finding that the model performs better for colon than for rectal cancer risk prediction is in line with previous studies [12, 25]. It has been considered clinically important to design one tool to predict both subtypes of cancer. However, it would be clinically advantageous if one could nuance the referrals based on risk, as the sigmoidoscopy used to diagnose distal colon cancer and rectal cancer is much less resource-demanding for the healthcare system and less burdensome for patients than the full colonoscopy.

On an overall level, the lifestyle-based LiFeCRC risk model has been suggested as a tool to identify the individuals most likely to benefit from lifestyle changes [12]. Several studies have reported a significant effect of changing the lifestyle on CRC prevention [31, 32]. However, the current model does not provide the patient or health practitioner with any information on which factors are most important to mitigate, or recommend any risk score cut-off for referral to diagnostic examinations such as endoscopy. Future studies should focus on establishing this link between the risk prediction model and clinical recommendations for lifestyle intervention and referral. It has also been suggested that future population-based studies focus on the added value of selected genetic variables and new biomarkers to established risk prediction models [6, 7].

A strength of the original LiFeCRC risk score is that it was developed based on a large European cohort study. Furthermore, it is a strength of our study that its performance is externally validated in the unselected, independent HUNT cohort which resembles the “average risk population”, which CRC screening programmes are designed for. As previous risk prediction models developed based on case–control studies have been criticised for estimates subject to recall bias, this is not an issue for the present study. Other strengths are the long follow-up time after participation in HUNT, accounting for the long latency period of CRC development, and the completeness of end-of-follow-up data by the CRN.

The modified LiFeCRC risk prediction model has some limitations. Although we state that this risk score may be used in an unselected, asymptomatic, European population, it is not applicable to patients with Crohn’s disease, ulcerative colitis, familial adenomatous polyposis, hereditary nonpolyposis CRC or other conditions with an intrinsic high risk of CRC [12]. However, it is already established that the abovementioned high-risk population needs other follow-up than the standard CRC screening programme. In addition, particularly due to differences in diet, the modified LiFeCRC score needs to be validated in other countries, preferably also outside Europe. Furthermore, patients diagnosed with CRC prior to the measurement of exposure in HUNT2/HUNT3 were excluded from the present study, and if these patients were characterised by certain risk factors (i.e. heavy smoking), selection bias would occur. If such, the risk estimates would be more modest than if these individuals were included. Finally, the full LiFeCRC score including all risk factors could only be calculated in 32,738 of the 78,580 participants due to missing values on food consumption. This illustrates that many study participants omit the questions about diet if they get too many and too detailed. Future studies should focus on keeping measurements of diet as simple as possible.

In conclusion, readily available clinical variables and food frequency questions can be used in a modified LiFeCRC risk score to identify patients at risk of CRC in Norway and may improve primary prevention by motivating to lifestyle change or participation in the CRC screening programme.

## Supplementary Information

Below is the link to the electronic supplementary material.

Supplementary file1 (PDF 779 KB)

## Data Availability

Data may be obtained from a third party and are not publicly available. The data that support the findings of this study are deidentified participant data, available from HUNT upon application (https://www.ntnu.no/hunt, e-mail: kontakt@hunt.ntnu.no). Restrictions apply to the availability of these data, which were used under licence for this study.

## Declarations

### Disclaimers

The study has used data from the Cancer Registry of Norway. The interpretation and reporting of these data are the sole responsibility of the authors, and no endorsement by the Cancer Registry of Norway is intended nor should be inferred.
