# Supplementary material for: External validation of the colorectal cancer risk score LiFeCRC using food frequency questions in the HUNT study
Source: Int J Colorectal Dis. 2024 Apr 25;39(1):57. doi: 10.1007/s00384-024-04629-4 (PMC11045582; doi:10.1007/s00384-024-04629-4)
Supplement: Supplementary file 1 — Supplementary file1 (PDF 779 KB) [file 384_2024_4629_MOESM1_ESM.pdf]

External validation of the LiFeCRC score and use of food frequency questions in the HUNT study

Brenne, Siv S. M.D.[a, b], Ness-Jensen, Eivind M.D., Ph.D.[b,c,d], , Laugsand, Eivor A. M.D., Ph.D.[a, b]

[a] Department of Surgery, Levanger Hospital, Nord-Trøndelag Hospital Trust, Levanger, Norway

[b] HUNT Research Centre, Department of Public Health and Nursing, NTNU, Norwegian University of Science and Technology, Levanger, Norway

[c] Department of Medicine, Levanger Hospital, Nord-Trøndelag Hospital Trust, Levanger, Norway

[d] Upper Gastrointestinal Surgery, Department of Molecular Medicine and Surgery, Karolinska Institutet, Karolinska University Hospital, Stockholm, Sweden

Correspondence: Siv S. Brenne, M.D., HUNT Research Centre, Department of Public Health and Nursing, NTNU, Norwegian University of Science and Technology, Forskningsveien 2, N-7600 Levanger, Norway. E-mail: siv.s.brenne@ntnu.no. ORCID: https://orcid.org/0000-0002-6227-8388

Telephone: +47 74075180, Fax: +47 74098500

Supplementary Table 1

Risk factor	M/Q	Definition	In CRC risk calculator
Age	M	Continuous: Age in years based on date of birth incorporated in the unique national personal identification number	Aleksandrova[1], Wells[2] Freedman[3], Hong[4]: continuous<br>Driver[5]: 40-49, 50-59, 60-69, ≥ 70<br>Ma[6]: 40-44, 45-49, 50-54, 55-59, 60-64, 65-69<br>Guo[7]: < 40, 40-49, 50-59, ≥60<br>Chen[8]: 40-49, 50-59, 60-69, >69<br>Betes[9]: ≤50, 51-60, 61-70, >70<br>Imperiale[10]: 50-54, 55-59, 60-64, 65-69, ≥ 70<br>Tao[11]: per 5 year increment
Sex	M	Binary: Based on the national personal identification number	Aleksandrova[1], Chen[8], Betes[9], Imperiale[10], Wells[2], Freedman[3], Tao[11], Hong[4]
Weight	M	Continuous: Weight in kilograms	Aleksandrova[1], Driver[5], Ma[6], Guo[7], Chen[8], Betes[9], Hong[4], Imperiale[10], Wells[2], Freedman[3], Tao[11]: not included
Height	M	Continuous: Height in centimeters	Aleksandrova[1]: continuous<br>Colditz[12]: continuous 6 inch increment<br>Hong[4], Driver[5], Ma[6], Guo[7], Chen[8], Betes[9], Imperiale[10], Wells[2], Freedman[3], Tao[11]: not included
Waist circumference	M	Continuous: Waist circumference in centimeters	Aleksandrova[1]: continuous<br>Guo[7]: <95 or ≥95 for men<br>Hong[4], Driver[5], Ma[6], Chen[8], Betes[9], Imperiale[10], Wells[2], Freedman[3], Tao[11]: not included
Body mass index (BMI)	C	Continuous: Weight in kilograms divided by the squared height in meters	Aleksandrova[1], Wells[2], Freedman[3]: continuous<br>Driver[5]: <25, 25-29.9, ≥30<br>Ma[6]:< 25, ≥ 25<br>Guo[7]: <18.5, 18.5-24.9, 25.0-29.9, ≥30<br>Betes[9]: ≤25, 26-30, >30<br>Colditz[12]: > 27 vs < 21<br>Wei[13]: ≤ 23.9, ≥24<br>Chen[8], Imperiale[10], Tao[11], Hong[4]: not included
Diabetes mellitus	Q	Binary: "Do you or have you ever had diabetes?" answered by "yes" or "no".	Aleksandrova[1]: excluded<br>Driver[5], Guo[7], Wells[2]: yes or no<br>Ma[6], Hong[4], Chen[8], Betes[9], Imperiale[10], Freedman[3], Tao[11]: not included

Alcohol intake	Q	**HUNT2** "Are you totally abstinent from alcohol?" answered by yes or no. "How many times a month do you drink alcohol?" answered by a number where ≥4 coded as "≥once/week".<br>**HUNT3** "During the last 12 months, how often have you been drinking alcohol?" answered by "totally abstinent or less than monthly".<br>or "<once/week but ≥monthly" or "≥once/week"<br>Binary: "≥once/week" vs less	Aleksandrova[1]: daily (yes or no)<br>Driver[5]: weekly (yes or no)<br>Ma[6]: never, occasional, <300g/week, ≥ 300g/week<br>Guo[7]: monthly for more than 6 months (yes or no)<br>Imperiale[10]: men ≥ 11/week, women ≥6/week<br>Wells[2]: drinks pr day continuous<br>Colditz[12]: >1 drink/day yes or no<br>Hong[4]: once a week, 2-3/month, 1-2/week, 3-4/week, 5-6/week, everyday<br>Tao[11]: ethanol g/day (pr 10g increment)<br>Chen[8], Betes[9], Freedman[3]: not included
Smoking status	Q	Binary:<br>Yes = 1 = Current or former smoker<br>No = 0 = Never smoker	Aleksandrova[1], Driver[5]: yes (current/former) or no<br>Ma[6]: yes (current) or no (former/never)<br>Guo[7]: never, former, current<br>Wei[13]: non, former, current<br>Hong[4]: duration in years<br>Chen[8], Betes[9]: not included
Smoking packyears	C	Continuous: Packyears is packs of cigarettes per day multiplied by the years smoked	Imperiale[10]: <0-<30 or ≥ 30 packs/year<br>Wells[2]: continuous<br>Colditz[12]: ≥ 25 cig/day vs none<br>Guo[7]: (at least 1 cig/day for >6months) yes or no<br>Hong[4]: duration in years<br>Tao[11]: per 10 packyears increment<br>Freedman[3]: cigs/day
Physical activity, Metabolic equivalents (MET) hours pr week	Q/C	Continuous: METs and MET hours/week were calculated as in previous HUNT-publications<br><br>Binary: individuals were categorized as physically active or not.<br><br>To align with LiFe CRC score:<br>Yes = 1 = > 8.3 MET-h/week<br>No = 0 = ≤ 8.3 MET-h/week	Aleksandrova[1]: physically active (yes or no)<br>Ma[6]: MET hours/day < 24.7, 24.7-<34.6, ≥ 34.6<br>Imperiale[10]: regular yes/no, moderate hours/week, vigorous 1h/week yes/no<br>Driver[5]: rarely (yes or no)<br>Wells[2]: moderate activity pr day (hours) continuous<br>Freedman[3]: leisure time activity h/week<br>Chen, Betes[9], Tao[11]: not included
Vegetables	Q	"How often do you normally eat this type of food?" answered by "0-3 times per month", "1-3 times per week", "4-6 times per week", "once a day" or "two or more times per day".<br>To align with LiFeCRC score (one serving is 100 g):	Aleksandrova[1]: in 100 g/day.<br>Imperiale[10]: (servings pr week) ≤3, 4-5, ≥ 6<br>Colditz[12]: continuous<br>Freedman[3]: serv/day

		0-3 times pr month → 0<br>1-3 times pr week → 0,3<br>4-6 times pr week → 0,6<br>Once a day → 1<br>Two or more times a day → 2<br><br>Binary: < daily intake vs. more	Driver[5], Ma[6], Guo[7], Chen[8], Betes[9], Hong[4], Wells[2], Tao[11]: not included
Dairy products (Milk)	Q	"How often do you drink milk?" answered by "never", "<1 glass/day", "1-3 glasses/day" or ">3 glasses/day"<br><br>To align with LiFeCRC score (1 glass is 200 grams):<br>Never →0<br>< 1 glass/day → 0,5 x2 = 1<br>1-3 glass/day → 2 X2 = 4<br>4 glasses or more →4 X 2 =8<br><br>Binary: < 1 glass/day vs. more	Aleksandrova[1]: in 100 g/day.<br>Driver[5], Ma[6], Guo[7], Chen[8], Betes[9], Hong[4], Imperiale[10], Wells[2], Freedman[3], Tao[11]: not included
Processed meat	Q	"How often do you normally eat this type of food (sausage/hamburger)?" answered by "0-3 times per month", "1-3 times per week", "4-6 times per week", "once a day" or "two or more times per day".<br><br>To align with LiFeCRC score (1 sausage or hamburger is 100 g):<br>0-3 times pr month → 0<br>1-3 times pr week → 0,3 x2 → 0,6<br>4-6 times pr week → 0,6x2 → 1,2<br>Once a day → 1x2→2<br>Two or more times → 2x2→4<br><br>Binary: ≥ daily intake vs less	Aleksandrova[1]: in 50 g/day.<br>Driver[5], Ma[6], Guo[7], Chen[8], Betes[9], Hong[4], Imperiale[10], Wells[2], Freedman[3], Tao[11]: not included
Sugar and confectionary	Q	"How often do you normally eat this type of food (chocolate/candy)?" answered by "0-3 times per month", "1-3 times per week", "4-6 times per week", "once a day" or "two or more times per day".<br><br>To align with LiFeCRC score (1 chocolate of 100 g is 50 g sugar):	Aleksandrova[1]: in 50 g/day.<br>Driver[5], Ma[6], Guo[7], Chen[8], Betes[9], Hong[4], Imperiale[10], Wells[2], Freedman[3], Tao[11]: not included

		0-3 times pr month → 0<br>1-3 times pr week → 0,3 x2 → 0,6<br>4-6 times pr week → 0,6x2 → 1,2<br>Once a day → 1x2→2<br>Two or more times → 2x2→4<br><br>Binary: ≥ daily intake vs less	
Bread	Q	"What type of bread do you usually eat?" answered by "white/white multigrain/semi wholegrain" or "wholegrain/dark/crispbread"<br><br>Categorical: dark/crisp vs. other	Aleksandrova[1], Driver[5], Ma[6], Guo[7], Chen[8], Betes[9], Hong[4], Imperiale[10], Wells[2], Freedman[3], Tao[11]: not included
Fruit/berries	Q	"How often do you normally eat this type of food?" answered by "0-3 times per month", "1-3 times per week", "4-6 times per week", "once a day" or "two or more times per day".<br><br>Categorical: < daily intake vs. more	Aleksandrova[1], Driver[5], Ma[6], Guo[7], Chen[8], Betes[9], Hong[4], Imperiale[10], Wells[2], Freedman[3], Tao[11]: not included
Fish	Q	"How often do you normally eat this type of food?" answered by "0-3 times per month", "1-3 times per week", "4-6 times per week", "once a day" or "two or more times per day".<br><br>Categorical: < daily intake vs. more	Aleksandrova[1], Driver[5], Ma[6], Guo[7], Chen[8], Betes[9], Hong[4], Imperiale[10], Wells[2], Freedman[3], Tao[11]: not included

Abbreviations: BMI, body mass index; M, measurement; Q, questionnaire, C, calculated

References

1. Aleksandrova K, Reichmann R, Kaaks R, et al. Development and validation of a lifestyle-based model for colorectal cancer risk prediction: the LiFeCRC score. BMC Med 2021;19:1.
2. Wells BJ, Kattan MW, Cooper GS, et al. Colorectal cancer predicted risk online (CRC-PRO) calculator using data from the multi-ethnic cohort study. J Am Board Fam Med 2014;27:42-55.
3. Freedman AN, Slattery ML, Ballard-Barbash R, et al. Colorectal cancer risk prediction tool for white men and women without known susceptibility. J Clin Oncol 2009;27:686-93.

4. Hong SN, Son HJ, Choi SK, et al. A prediction model for advanced colorectal neoplasia in an asymptomatic screening population. PLoS One 2017;12:e0181040.
5. Driver JA, Gaziano JM, Gelber RP, et al. Development of a risk score for colorectal cancer in men. Am J Med 2007;120:257-63.
6. Ma E, Sasazuki S, Iwasaki M, et al. 10-Year risk of colorectal cancer: development and validation of a prediction model in middle-aged Japanese men. Cancer Epidemiol 2010;34:534-41.
7. Guo L, Chen H, Wang G, et al. Development of a risk score for colorectal cancer in Chinese males: A prospective cohort study. Cancer Med 2020;9:816-23.
8. Chen G, Mao B, Pan Q, et al. Prediction rule for estimating advanced colorectal neoplasm risk in average-risk populations in southern Jiangsu Province. Chin J Cancer Res 2014;26:4-11.
9. Betés M, Muñoz-Navas MA, Duque JM, et al. Use of Colonoscopy As A Primary Screening Test for Colorectal Cancer in Average Risk People. Official journal of the American College of Gastroenterology | ACG 2003;98:2648-54.
10. Imperiale TF, Monahan PO, Stump TE, et al. Derivation and validation of a predictive model for advanced colorectal neoplasia in asymptomatic adults. Gut 2021;70:1155-61.
11. Tao S, Hoffmeister M, Brenner H. Development and validation of a scoring system to identify individuals at high risk for advanced colorectal neoplasms who should undergo colonoscopy screening. Clin Gastroenterol Hepatol 2014;12:478-85.
12. Colditz GA, Atwood KA, Emmons K, et al. Harvard report on cancer prevention volume 4: Harvard Cancer Risk Index. Risk Index Working Group, Harvard Center for Cancer Prevention. Cancer Causes Control 2000;11:477-88.
13. Wei YS, Lu JC, Wang L, et al. Risk factors for sporadic colorectal cancer in southern Chinese. World J Gastroenterol 2009;15:2526-30.

Supplementary Table 2. Cox regression with colorectal cancer prediction variables

		Both sexes			Men			Women	
Possible predictors	HR	95% CI	p	HR	95% CI	p	HR	95% CI	p
Age (in years)	1.08	1.07-1.09	**< 0.001**	1.08	1.07-1.10	**< 0.001**	1.08	1.06-1.09	**< 0.001**
Waist circumference (in cm)	1.01	1.00-1.02	**0.010**	1.02	1.01-1.03	**0.008**	1.01	1.00-1.02	0.294
Height (in cm)	1.02	1.00-1.03	**0.006**	1.00	0.98-1.02	0.880	1.02	1.00-1.05	0.104
Alcohol ≥ once a week (reference < weekly)	1.23	1.00-1.50	**0.045**	1.22	0.94-1.59	0.133	1.20	0.88-1.64	0.251
Smoking, ever (reference never)	1.33	1.09-1.62	**0.005**	1.18	0.90-1.55	0.235	1.42	1.06-1.91	**0.020**
Physically active (reference ≤ 8.3 MET-h/week)	0.93	0.76-1.13	0.449	1.03	0.79-1.35	0.807	0.79	0.59-1.06	0.118
Vegetables intake (increasing frequency)	1.04	0.83-1.31	0.748	1.22	0.88-1.68	0.240	0.93	0.66-1.30	0.655
Milk intake (increasing frequency)	0.98	0.94-1.03	0.423	0.98	0.93-1.04	0.485	0.98	0.92-1.05	0.617
Processed meat intake (increasing frequency)	1.33	0.98-1.80	0.070	1.38	0.95-2.03	0.095	1.19	0.72-1.99	0.496
Sugar and confectionary (increasing frequency)	0.86	0.72-1.03	0.103	0.87	0.69-1.11	0.262	0.82	0.62-1.10	0.183

cm centimeters, *HR* hazard ratio, *CI* confidence interval, *MET-h/week* metabolic equivalent hours/week. p-values from multivariable cox regression analyses

Supplementary Table 3. Cox regression with colon cancer prediction variables

		Both sexes			Men			Women		
Possible predictors		HR	95% CI	p	HR	95% CI	p	HR	95% CI	p
Age	(in years)	1.09	1.08-1.10	**< 0.001**	1.09	1.07-1.11	**< 0.001**	1.09	1.07-1.11	**< 0.001**
Waist circumference	(in cm)	1.02	1.01-1.03	**0.004**	1.02	1.00-1.03	**0.023**	1.01	1.00-1.03	0.057
Height	(in cm)	1.02	1.00-1.03	**0.026**	1.00	0.98-1.03	0.757	1.02	0.99-1.05	0.219
Alcohol ≥ once a week	(reference < weekly)	1.26	1.00-1.61	0.055	1.22	0.89-1.67	0.208	1.31	0.90-1.91	0.161
Smoking, ever	(reference never)	1.36	1.07-1.72	**0.012**	1.26	0.91-1.75	0.163	1.41	0.99-2.01	0.060
Physically active	(reference ≤ 8.3 MET-h/week)	0.90	0.71-1.13	0.365	0.98	0.71-1.35	0.905	0.80	0.56-1.13	0.202
Vegetables intake	(increasing frequency)	0.97	0.73-1.28	0.829	1.24	0.84-1.82	0.280	0.77	0.51-1.17	0.221
Milk intake	(increasing frequency)	1.01	0.95-1.06	0.857	1.00	0.93-1.07	0.916	1.01	0.94-1.10	0.748
Processed meat intake	(increasing frequency)	1.28	0.89-1.84	0.192	1.42	0.90-2.23	0.129	1.06	0.57-1.96	0.862
Sugar and confectionary	(increasing frequency)	0.87	0.70-1.08	0.205	0.79	0.59-1.07	0.130	0.96	0.70-1.31	0.785

cm centimeters, *HR* hazard ratio, *CI* confidence interval, *MET-h/week* metabolic equivalent hours/week. p-values from multivariable cox regression analyses

Supplementary Table 4. Cox regression with rectal cancer prediction variables

		Both sexes			Men			Women		
Possible predictors		HR	95% CI	p	HR	95% CI	p	HR	95% CI	p
Age	(in years)	1.06	1.05-1.08	**< 0.001**	1.07	1.04-1.09	**< 0.001**	1.05	1.03-1.08	**< 0.001**
Waist circumference	(in cm)	1.00	0.99-1.02	0.742	1.02	0.99-1.04	0.147	0.99	0.97-1.01	0.347
Height	(in cm)	1.02	1.00-1.04	0.090	0.99	0.95-1.02	0.454	1.03	0.98-1.08	0.237
Alcohol ≥ monthly	(reference < monthly)	1.16	0.81-1.67	0.410	1.24	0.77-2.00	0.377	1.01	0.58-1.77	0.973
Smoking, ever	(reference never)	1.30	0.91-1.85	0.157	1.02	0.63-1.66	0.943	1.50	0.88-2.56	0.135
Physically active	(reference ≤ 8.3 MET-h/week)	1.00	0.70-1.43	0.989	1.17	0.71-1.93	0.536	0.78	0.46-1.33	0.364
Vegetables intake	(increasing frequency)	1.21	0.81-1.81	0.351	1.17	0.64-2.12	0.611	1.35	0.77-2.35	0.293
Milk intake	(increasing frequency)	0.93	0.86-1.01	0.083	0.94	0.85-1.05	0.264	0.92	0.81-1.04	0.161
Processed meat intake	(increasing frequency)	1.45	0.83-2.52	0.189	1.31	0.64-2.66	0.462	1.57	0.65-3.78	0.314
Sugar and confectionary	(increasing frequency)	0.82	0.58-1.16	0.265	1.05	0.72-1.54	0.801	0.48	0.25-0.94	**0.032**

cm centimeters, *HR* hazard ratio, *CI* confidence interval, *MET-h/week* metabolic equivalent hours/week. p-values from multivariable cox regression analyses
